# Recent Trends in the Effect of Race and Gender on the Orthopedics Match

**DOI:** 10.7759/cureus.53247

**Published:** 2024-01-30

**Authors:** Zachary Brandt, Jun Ho Chung, Jacob Razzouk, Montri D Wongworawat

**Affiliations:** 1 Department of Orthopaedic Surgery, School of Medicine, Loma Linda University, Loma Linda, USA; 2 Department of Orthopaedic Surgery, Loma Linda University Medical Center, Loma Linda, USA

**Keywords:** diversity, orthopedic match, ethnicity, gender, race

## Abstract

Introduction

Orthopedics has a reputation for being competitive but not diverse. Leaders of the orthopedics community have been making efforts to increase the participation of women and minorities in the field by raising awareness and strengthening pipeline programs. We aim to explore the trends in the risk of not matching by comparing the proportions of women and underrepresented populations in the applicant pools versus proportions in residency programs. Simultaneously, we aim to evaluate if women or underrepresented population applicants exhibit a lower likelihood of applying to orthopedics compared to male and White applicants.

Methods

The study received an IRB exemption. The authors collected Accreditation Council for Graduate Medical Education (ACGME) data books for the years 2015-2016 to 2022-2023 to obtain demographic information on orthopedic residents in training during each of those academic years. The pool of corresponding applicants (for example: residents in training during the 2021-2022 academic year would consist of five classes, made up of applicants from 2016-2017 to 2020-2021) was then tabulated from Electronic Residency Applications Service (ERAS) statistics, which are publicly available on the Association of American Medical Colleges (AAMC) website. The race and gender composition of the applicant pool was compared to that of corresponding enrolled residents to calculate the relative risk (RR) of women not matching compared to men and underrepresented population applicants (Blacks, Hispanics, Asians, and Native Americans) compared to White applicants. ERAS data was subsequently used to calculate the percentages of each demographic applying to all residency programs and orthopedic programs.

Results

For female applicants into orthopedic residency, they had a similar RR of going unmatched when compared to their male counterparts. In the academic year 2020-2021, there was an exception to this as women had a slightly higher RR of going unmatched. All underrepresented populations had a higher risk of not matching compared to White applicants for all cycles, peaking for residents in training in 2020-2021. The trendline improved for residents in the following year. Throughout the study, women accounted for 46.61% of applicants applying for any residency; however, they only accounted for 16.98% of applicants applying for orthopedic residency. A similar discrepancy is noted among Asian applicants but not Black or Hispanic applicants.

Conclusions

Underrepresented populations were increasingly less likely to match into orthopedics relative to White applicants until 2021. In the academic year of 2021-2022, there was an improvement in this trend for all studied underrepresented populations. Although the exact explanation for this is unclear, it is associated with the transition to virtual applicant interactions. The female gender did not appear to be a consistent advantage or disadvantage in the match. Women and Asian applicants were less likely to apply to orthopedics than other specialties.

## Introduction

As diversity and its positive effects on patient care come under the increasing spotlight in various medical specialties, orthopedic surgery has gained a reputation in the medical community as a specialty that is highly competitive but also less accessible to women and underrepresented populations. Underrepresented populations have been defined as those of a disproportionately low number of individuals in the orthopedics workforce [1]. This includes applicants and residents who are self-described as Black, Hispanic, Asian, or Native on Accreditation Council for Graduate Medical Education (ACGME) and Association of American Medical Colleges (AAMC) surveys. Orthopedics has the lowest applicant match rates even among the most competitive specialties, with the ratio of applicants to the number of available spots growing each year [2]. Between 2006 and 2014, ethnic minorities across the board accounted for a comparably small proportion (only 25.6%) of all orthopedic surgery trainees [3]. During the same period, women made up 10.9%-14.4% of orthopedic trainees, which was a small proportion especially when compared to other surgical specialties [3].

Such a low proportion of ethnic minorities and women in orthopedics has brought on not only criticism but also scrutiny of the orthopedics match process. Research by Poon et al. showed that minority applicants match into orthopedics at lower rates compared to their Caucasian counterparts despite having competitive class rankings, test scores, and extracurricular activities [4]. There are many residency programs in the nation with no underrepresented minorities, and the number was shown to have increased between 2003 and 2017 [5]. Knowledge of such discrepancies has caused many leading figures in the orthopedic surgical field to explore ways to increase women and minority participation. Several ways to do this include forming pipeline programs starting early in the medical career, establishing organizations such as the American Association of Latino Orthopaedic Surgeons, or introducing “fitness” as a metric in the match process [5,6].

After such measures have been put in place, not much is known about their impact on the trends of the orthopedics match process. The most recent literature has a focus on cross-sectional studies and describes the need for up-to-date information on how race and gender may affect residency applications [7,8]. Furthermore, recent years saw dramatic changes in the traditional match process due to the COVID-19 pandemic, which caused significant portions of the interview and audition process to be done in a virtual setting. The authors explored changes and trends in the relative risk (RR) of minority and women applicants not matching into orthopedics alongside the demographic makeup of orthopedic applicants compared to all resident applicants.

This article was previously presented as a meeting abstract at the Orthopedic Summit Meeting on September 22, 2023.

## Materials and methods

The study received an IRB exemption. The data used in this study is sourced from two different data banks, the ACGME data resource book and the Electronic Residency Application Service (ERAS) data sheets. ACGME data on residents, also known as applicants who matched, encompasses all years of residents within that academic year, including postgraduate years (PGY) one through five for orthopedic residents. ERAS data sheets, on the other hand, provide annual demographic data on residency applicants. Both of these data resources provide population data, including all applicants (ERAS) and residents (ACGME) with their respective demographic information.

Using data from the ACGME data resource book, this study examined the demographic composition of residents in training over eight academic years from 2015-2016 to 2022-2023. The authors also utilized information from the ERAS data sheets from match years 2011 to 2022 to determine the demographic characteristics of the corresponding applicant pools during those years. The additional years of ERAS data sheets were needed as applicants in the 2011 cycle would be PGY-5 in the 2015-2016 academic year. Specifically, the two data banks were used to calculate the proportion of applicants from different demographic backgrounds who successfully matched and became residents.

For instance, to analyze residents in training during the academic year 2021-2022, the study considered applicants who matched from 2017 to 2021. The researchers compared the demographics of applicants from the five ERAS corresponding data sheets with the residents during the 2021-2022 academic year using the 2021-2022 ACGME data resource book and calculated the RR of not matching for underrepresented populations compared to White applicants and the RR for female applicants compared to male applicants. When comparing underrepresented populations to White applicants, calculations included both the male and female genders. Calculations comparing the RR of going unmatched for females versus males included all races and ethnicities. Underrepresented population applicants include Black, Hispanic, Asian, and native applicants. This calculation was performed for the academic years from 2015-2016 to 2022-2023. SPSS version 28 (IBM Corp., Armonk, NY) was utilized for all statistical analyses with alpha defined as p < 0.05. Descriptive statistics consisted of standard deviations (SD) and 95% confidence intervals (CI).

To ensure consistency, all racial and ethnic designations were standardized across the data sheets, except for the Hispanic ethnic designation for the applicant years 2011 and 2012. For these years, race and ethnicity were recorded separately on ERAS data, prompting the authors to extract the Hispanic ethnicity designation specifically for applicants who self-identified as Hispanic.

Using additional annual ERAS data provided on the AAMC website, the number and demographics of all applicants into any residency were collected. These numbers were totaled for the years studied. The percentage of each demographic was subsequently calculated for all applicants. This same technique was used on applicants for orthopedic residency. The demographic makeup of applicants to all residency programs was then compared to those of applicants to orthopedic residency.

## Results

In the academic year 2020-2021, women were seen to have a slightly higher relative risk of not matching compared to men (RR 1.08 [95% CI: 1.00-1.15]; p = 0.038, Table 1), a disadvantage that was not seen again as all other relative risk values did not meet statistical significance (p-value > 0.05). Values for all application years are displayed in Table 1.

**Table 1:** Relative risk of not matching for women applicants compared to male applicants Relative risk (95% confidence interval).

Residency year	Women	p-values
2015-2016	1.00 (0.93 to 1.07)	0.93
2016-2017	1.00 (0.93 to 1.07)	0.92
2017-2018	0.99 (0.92 to 1.06)	0.72
2018-2019	1.06 (0.99 to 1.14)	0.10
2019-2020	1.07 (0.99 to 1.15)	0.10
2020-2021	1.08 (1.00 to 1.15)	0.04
2021-2022	1.00 (0.94 to 1.07)	0.92
2022-2023	0.95 (0.89 to 1.01)	0.08

All underrepresented populations, including Native American, Asian, Hispanic, and Black, had a higher RR of not matching compared to White applicants across all academic years studied, from 2015-2016 to 2022-2023. From 2015-2016 to 2020-2021, the RR of not matching increased for all minorities. Native Americans in 2015-2016 had an RR of not matching at 1.67, while the RR of not matching was 2.91 in 2020-2021 (p-value < 0.05 for both). Asians had an RR of not matching, which was 1.38 in 2015-2016 and 1.54 in 2020-2021 (p-value < 0.05 for both). Hispanics' RR of not matching in 2015-2016 was 1.55, while it was 2.35 in 2020-2021 (p-value < 0.05 for both). Black applicants’ RR of not matching was 1.55 in 2015-2016 and 1.93 in 2020-2021 (p-value < 0.05 for both). All values are displayed in Table 2.

**Table 2:** Relative risk of not matching for underrepresented population applicants compared to White applicants Relative risk (95% confidence interval).

Residency year	Black	Hispanic	Asian	Native
2015-2016	1.55 (1.47 to 1.63)	1.55 (1.47 to 1.64)	1.37 (1.31 to 1.45)	1.67 (1.51 to 1.85)
2016-2017	1.52 (1.44 to 1.61)	1.55 (1.46 to 1.64)	1.35 (1.29 to 1.42)	1.70 (1.53 to 1.88)
2017-2018	1.50 (1.41 to 1.60)	1.53 (1.44 to 1.62)	1.38 (1.31 to 1.45)	1.74 (1.58 to 1.91)
2018-2019	1.57 (1.48 to 1.68)	1.59 (1.49 to 1.68)	1.41 (1.34 to 1.49)	1.88 (1.72 to 2.05)
2019-2020	1.83 (1.69 to 1.97)	1.92 (1.79 to 2.06)	1.64 (1.53 to 1.74)	2.26 (2.02 to 2.53)
2020-2021	1.93 (1.78 to 2.09)	2.35 (2.21 to 2.50)	1.54 (1.43 to 1.65)	2.91 (2.70 to 3.12)
2021-2022	1.72 (1.59 to 1.86)	1.47 (1.36 to 1.60)	1.49 (1.40 to 1.59)	2.67 (2.51 to 2.84)
2022-2023	1.56 (1.45 to 1.68)	1.34 (1.24 to 1.45)	1.38 (1.30 to 1.47)	2.41(2.28 to 2.55)

**Figure 1:**
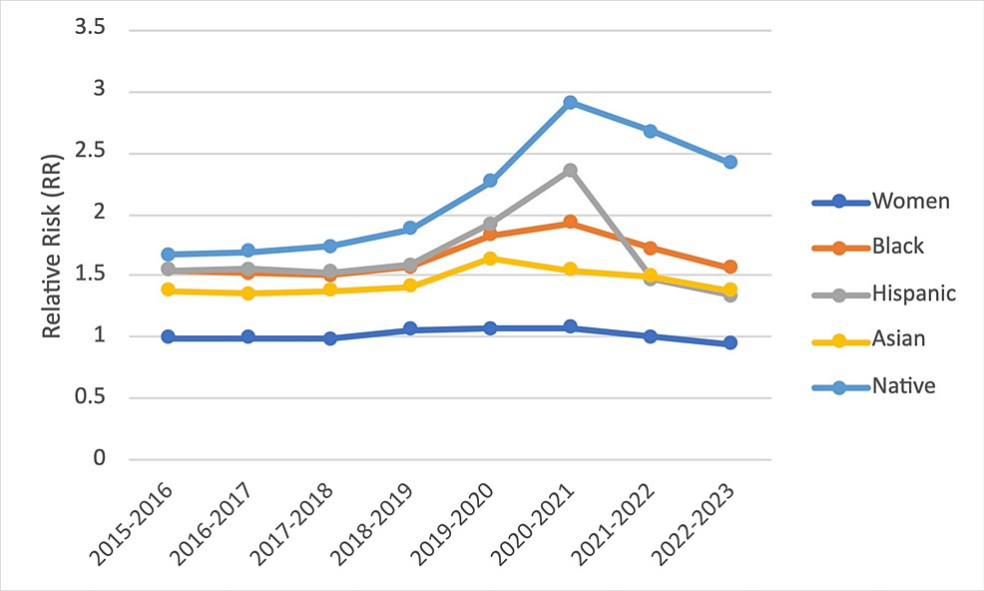
Trends in relative risk of not matching, underrepresented populations compared to White applicants, and women applicants compared to male applicants from 2015 to 2023 Listed years (x-axis) indicate the start of residency, not the year of the interview.

Although all underrepresented populations were still disadvantaged compared to White applicants in 2021-2022, their RR of not matching declined compared to the previous year. The change in RR for not matching from 2020-2021 to 2021-2022 was 1.93 to 1.72 for Blacks, 2.35 to 1.47 for Hispanics, 1.54 to 1.49 for Asians, and 2.91 to 2.67 for Native Americans (Table 2, p-value < 0.05 for all). This trend continued into the 2022-2023 academic year. A graph showing all RRs plotted over time is presented in Figure 1.

Women applicants accounted for 46.61% of applicants across all residencies while making up only 16.98% of applicants in orthopedics. A similar trend was observed in Asian applicants as they accounted for 23.66% of all applicants but only 15.80% of applicants in orthopedics. Hispanic, Black, and native applicants did not demonstrate this same trend. Hispanics accounted for 7.71% of all applicants and a similar 7.74% in orthopedics. Black applicants accounted for 7.13% of all residency programs and a similar 7.05% in orthopedic residency. Native applicants accounted for 0.52% of all residency applicants and a higher 0.82% in orthopedic residency applicants. All values are displayed in Table 3.

**Table 3:** Demographic distribution of applicants from 2011 to 2022

Residency	Women	Black	Hispanic	Asian	Native
All residency programs	46.61%	7.13%	7.71%	23.66%	0.52%
Orthopedics	16.98%	7.05%	7.74%	15.80%	0.82%

## Discussion

It is already common knowledge that men and Caucasians make up a significant majority of orthopedic surgeons and residents. Research done by Poon et al. demonstrated that belonging to a minor ethnic group such as Asian, Black, Hispanic, or Native American has been associated with lower odds of admission into orthopedics residencies [4]. However, the data used in Poon et al.’s research is based on resident classes from 2005 to 2014 and does not show trends in orthopedics match rates throughout the years [4]. As there have been subsequent efforts to increase the participation of women and minorities in orthopedics, the authors wanted to explore the up-to-date effects of these efforts in the orthopedics match rates in more recent years. Although the authors discovered that gender did not seem to have a consistent effect on the likelihood of matching, they found that even recently, being part of an underrepresented population group continued to be a worsening disadvantage for matching into orthopedics until the 2020-2021 academic year.

Women overall had a comparable risk of not matching to men. Although women had a relative disadvantage in matching in the academic year 2020-2021, such differences were minor, and the relative risks were not statistically significant for the other years studied. This is consistent with results from the study by Poon et al., which showed that gender did not appear to be a meaningful variable in the likelihood of matching into orthopedics residency between 2005 and 2014 [4]. However, this is not to say that women have an equal representation in orthopedics as men. Although the proportion of women in medical school has gradually increased to half of graduating students recently, women made up 14% of orthopedic surgery residents in the year 2016-2017 [9]. The takeaway from these results is that the lack of representation of women among orthopedic surgery residents is not due to a prejudiced match process but rather due to other variables that eventually affect women’s decision to apply in the first place. This was further demonstrated as women only made up 16.98% of applicants to orthopedic residency. Research by Rao et al. has suggested that women tend to be less interested in orthopedics than men [10]. Some suggested reasons include a lack of role models, little exposure during medical school, and negative stereotypes about the field [11,12]. Strategies to increase the participation of women in orthopedics may therefore be found in encouraging application by increased exposure to orthopedics during medical school and mentorship from role models [13].

The underrepresented populations of the study (Black, Hispanic, Asian, and Native Americans) were all at higher risk of not matching compared to White applicants, with Native American applicants being most likely to go unmatched. Despite ongoing efforts to strengthen pipeline programs for minority students and address bias in the application process, the apparent disadvantage of minority applicants has been increasing, with all underrepresented populations less likely to match in 2020-2021 compared to 2015-2016. When it comes to explaining the relatively low representation of minorities in orthopedics, the application and match process is a major hurdle. Compared to White applicants in the 2020-2021 application cycle, underrepresented populations had lower step scores while having more research projects [14]. However, even when controlling for variables such as STEP 1 and 2 scores, AOA status, etc., minorities were still less likely to match orthopedics compared to Caucasian applicants [4]. Research by Webber et al. suggests that White applicants may be implicitly favored by admission committees, who assign higher scores to applications from Whites when applicants’ race is disclosed to them [14].

The difference in the representation of underrepresented populations among orthopedic residents cannot be explained in the same way as female underrepresentation. The proportion of those applying to orthopedics from underrepresented groups is comparable to the proportion of applicants applying to all residencies. Black applicants accounted for 7.13% of applicants into all residency programs while also accounting for 7.05% of applicants into orthopedics. A similar trend is observed in both Hispanic and Native American applicants with the only exception being Asian applicants. This suggests a disparity with the application process and not a pipeline issue as it is for female applicants in orthopedics.

In the 2021-2022 academic year, underrepresented population applicants’ disadvantage decreased compared to White applicants in the match process. All underrepresented populations of the study were more likely to match in 2021-2022 compared to 2020-2021. Although numerous possible reasons could explain such a phenomenon, it should be noted that residents in training during 2021-2022 were the first group to include a class of residents who auditioned and interviewed on a virtual basis due to the COVID-19 pandemic. This theory is further supported by a continued decrease in the RR of going unmatched among all underrepresented populations in the academic year 2022-2023. Residency applications to match in 2021 were heavily affected by the pandemic, which initiated a national quarantine in March 2020, preventing students from rotating and interviewing in person [15]. Away rotations and interviews are the key components affecting an applicant’s likelihood to match as medical students can use the opportunities to build relationships with advocates and demonstrate traits that make them attractive to residency programs such as work ethic and surgical skills [16,17]. Away rotations are a strong independent predictor of matching to orthopedics, and 36% of applicants match where they auditioned [18,19]. At the same time, traditional in-person rotations and interviews can allow for the introduction of bias due to the associated financial costs. During the 2015 match cycle, applicants spent $3,656 on average on orthopedic residency interviews for traveling and housing [20]. Considering that underrepresented minority medical students are more likely to graduate medical school with debt, high price tags that come with in-person interviews and away rotations place uneven burdens on minority applicants while they navigate one of the most crucial steps for the match [18]. The most recent application cycle provides valuable insight into ways to increase the representation of underrepresented populations in orthopedics. In addition to virtual interviews, offering away rotation scholarships to underrepresented populations may offset not only the financial stress of orthopedics applications but also even the playing ground in favor of those who also happen to be more budget-constrained [18].

Limitations

There are a variety of limitations associated with the study. The study data cannot account for possible confounders such as AOA status, class rank, and school reputation, which also affect the match process. Additionally, findings are a moving average of five years as single-year data is unavailable. Therefore, the relative likelihood of going unmatched is calculated for all the residents of a single academic year rather than all the applicants for a certain application cycle. Furthermore, we could not adjust for irregular training schedules outside of the five-year requirement such as those who make multiple attempts to match into orthopedics or residents who take research years during training. The moving average of five years functions well as a surrogate data source as the rate of attrition from orthopedic programs has been cited to be less than 1% [21]. Regardless, the study provides valuable follow-up information on the more recent trends in the orthopedics match.

## Conclusions

Women applicants overall appear to have a similar likelihood of matching into orthopedics as male applicants. The underrepresentation of women in orthopedics is likely due to a discrepancy in the number of applicants and not inherited bias within the application process. Underrepresented populations appear to have a disadvantage compared to their White counterparts, with the trend worsening until 2021 despite initiatives to increase minority representation. In 2021, there was an improvement in this trend for all studied underrepresented populations. Although the exact explanation for this phenomenon is unclear, it is possibly affected by the virtual nature of interviews and rotations during the 2020-2021 application and match cycle. This trend continued in the academic year 2022-2023 when interviews were still conducted virtually. Despite its limitations, the study reflects general shifts in orthopedic surgery match rates based on demographic data and could inspire future studies with more granular data.
